# Impact of Health Care Policies on Patient Portal Adoption and Engagement in US Hospitals: Trends From 2012 to 2024

**DOI:** 10.63116/AH.000000003

**Published:** 2026-07-24

**Authors:** Shannon H. Houser, Wei Lyu

**Affiliations:** University of Alabama at Birmingham

**Keywords:** health information technology, health policy, patient engagement, patient portals

## Abstract

**Background:**

Patient portals are essential tools for patient engagement, transparency, and communication in US health care. Federal health information technology (IT) policies, such as Meaningful Use, Promoting Interoperability, and the 21st Century Cures Act, have played a pivotal role in driving the adoption of these tools. Despite prior work on portal availability and use, longitudinal, hospital-level analyses aligned with major policy milestones remain limited. This study examines national trends in hospital adoption of patient portal functionalities from 2012 to 2024 and assesses alignment with federal health IT policy initiatives.

**Methods:**

We analyzed 2012-2024 data from the American Hospital Association Annual Survey Information Technology Supplement. Three functional domains were evaluated: (1) patient access to information (view, download, and amend); (2) online administrative tools (bill payment, appointment scheduling, and prescription refill); and (3) patient-provider communication (secure messaging, patient-generated data submission, and external record import). Annual adoption rates were calculated, and monotonic trends were assessed using the Mann-Kendall test.

**Results:**

By 2024, 92% of hospitals had adopted both viewing and downloading functionalities (τ = 0.94 and τ = 0.97; *P* < .001). Bill payment increased from 45.9% (2012) to 87.0% (2020). By 2020, prescription refill (74.6%) exceeded appointment scheduling (66.5%). Secure messaging increased from 28.2% (2012) to 88.7% (2024), and patient-generated data submission from 8.4% to 67.7%. In contrast, amending information and importing external records showed greater volatility, with no significant monotonic trends over their measurement windows.

**Conclusions:**

US hospitals have made substantial progress in adopting patient portal functionalities over the past decade, broadly paralleling federal policy developments. Core access tools (view/download) are now nearly universal, whereas administrative and communication features continue to expand. Uneven uptake of advanced capabilities highlights variability in implementation and underscores the need for sustained policy support, technical assistance, and equity-focused strategies to ensure that all hospitals—and patients—realize the full benefits of digital engagement.

## Introduction

Over the past decade, US health care has undergone a rapid shift toward digitization, with patient portals emerging as central tools for patient engagement (PE), transparency, and communication between patients and providers. Patient portals offer secure access to medical records, test results, medication lists, and educational materials while also enabling functions such as secure messaging and prescription refills. Despite their promise, portal adoption and use have varied across hospitals and populations and are influenced by organizational and technological factors.^1^^,^^2^ Federal health information technology (IT) initiatives and regulatory policies have further accelerated the adoption and expansion of patient portal functionalities across US hospitals.

Several federal health IT policies have played a central role in driving patient portal adoption. The Health Information Technology for Economic and Clinical Health (HITECH) Act of 2009 and the Meaningful Use (MU) program incentivized hospitals to provide patients with timely electronic access to health information.^3^^,^^4^ Requirements expanded across MU Stages 1–3, leading to widespread portal deployment. In 2018, the program transitioned to Promoting Interoperability (PI), which emphasized interoperability and patient access.^5^ More recently, the 21st Century Cures Act, with information-blocking provisions implemented in 2021, required hospitals to provide patients with access to clinical notes and other electronic health information through portals and application programming interfaces (APIs).^6^^,^^7^ Together, these regulations made patient portals integral to hospital operations and PE strategies.

Hospital adoption of electronic health records and patient portals has grown substantially under these policies. By 2014, nearly all nonfederal acute care hospitals had adopted certified electronic health records (EHRs),^8^ and by the late 2010s, reports suggested that approximately 90% of health care organizations offered patient portal access to patients.^9^ From 2021 to 2024, the proportion of hospitals reporting Fast Healthcare Interoperability Resources-based application access for PE in inpatient settings increased from 56% to 69%.^10^ Smaller facilities have also made notable progress, with critical access hospitals expanding advanced PE functionalities from 18% in 2014 to 49% in 2023.^11^ Nevertheless, disparities persist, as rural and resource-limited hospitals continue to lag behind larger institutions. Recent descriptive analyses using multiple national data sources also document sustained growth in PE functionalities, reinforcing the broader trajectory of health IT adoption in US hospitals.^12^

Parallel to hospital adoption, PE has increased, particularly following recent regulatory changes. In 2022, approximately 3-quarters of individuals had been offered portal access, and more than half accessed their records, reflecting substantial increases compared with 2020.^13^ By 2024, 57% of individuals reported using patient portals or health applications, compared with 38% in 2020.^14^ Patients increasingly rely on portals to view test results, message providers, and access clinical notes, with more than 90% preferring immediate release of results, even when abnormal.^15^^,^^16^ However, disparities in portal use persist across race, income, education, and geographic location.^17^

Despite the well-documented increases in general patient portal availability, few studies have applied a longitudinal lens to evaluate trajectories across more than a decade of federal health IT policy implementation. The objective of this study is 2-fold: first, to examine national trends in hospital adoption of patient portal functionalities from 2012 to 2024 and, second, to describe how these trends align with key federal health IT policy milestones, including MU, PI, and the 21st Century Cures Act. By evaluating the evolution of specific tools over a decade, this study provides a comprehensive assessment of the effectiveness of federal health IT policy in fostering a digitally engaged patient population.

## Methods

### Data

We used data from the 2012-2024 American Hospital Association (AHA) Annual Survey Information Technology Supplement to examine changes in the adoption of PE functionalities over time. The AHA IT Supplement, completed annually by nearly half of US hospitals, collects information on health IT use and capabilities, including PE, health information exchange, EHR systems, and IT vendors. The survey is typically completed by a hospital’s chief information officer, and participation is voluntary. The instrument is periodically updated to capture the adoption of new health IT functionalities.

Annual hospital participation in the AHA IT Supplement varied across years. We reported the number of responding hospitals for each survey year (2012-2024) and calculated adoption rates using year-specific denominators. Analyses are limited to years in which each functionality was fielded, resulting in variation in analytic denominators across items as the survey instrument evolved over time. Comparable item-level data were not available for 2021; therefore, data from 2021 are excluded from all figures, tables, and trend analyses.

Our study sample included all US hospitals across the 50 states and the District of Columbia that submitted valid responses for a given year, yielding 39,514 hospital-year observations and an average of approximately 3000 hospitals surveyed annually. Because we used publicly available, deidentified, organization-level data, this analysis did not constitute human subjects research and did not require institutional review board approval.

### PE Measures

Because the PE module of the AHA IT Supplement evolved over time, we focused on functionalities consistently tracked across the study period. We organized these functionalities into 3 domains aligned with Table 1, which also summarizes relevant federal policy milestones and how they map to each domain.

- *Access:* Patient access to health information (view, download, and amend records).
- *Administration:* Online administrative services (appointment scheduling, bill payment, and prescription refills).
- *Exchange:* Patient-provider information exchange (secure messaging, submission of patient-generated health data [PGHD], and importation of external medical records).

**Table 1.** Major US Health IT Policy Milestones Aligned to Patient Portal Domains (2012-2024)

**Policy/regulation (issuing authority)**	**Time frame**	**Requirements and portal relevance**	**Primary portal domain(s) impacted**
HITECH Act (ARRA Title XIII) (HHS/CMS/ONC)	2009-2015 (incentive period); ongoing effects	Funded certified EHR adoption; established foundation for patient portals and electronic access that was later operationalized by MU^3^	Access; exchange
Meaningful Use Stage 1 (CMS/ONC)	2011-2013	Initiated electronic copies/after-visit summaries; early VDT capability^18^^,^^19^	Access
Meaningful Use Stage 2 (CMS/ONC)	2014-2016	Increased VDT thresholds; introduced secure patient-clinician messaging; advanced exchange^20^^,^^21^	Access; exchange
Meaningful Use Stage 3 (CMS/ONC)	2017-2018	Emphasized API-based patient access (USCDI data); strengthened patient engagement (secure messaging, patient-generated health data)^22^^,^^23^	Access; exchange
Promoting Interoperability (CMS/ONC)	2018-present	MU rebranded; standardized APIs and interoperability measures to support real-time patient access across settings^24^	Access; exchange
21st Century Cures Act + ONC Cures Final Rule (Congress/ONC)	2016-present	Information-blocking prohibitions; ONC certification requirements supporting standardized FHIR-based APIs; expanded USCDI data classes for patient access and sharing.^6^^,^^25^	Access; exchange
ONC Certification and FHIR-Based API Standards (ONC/HL7)	2015-present	Supports standardized API-based access to patient data, enabling apps and broader portal ecosystems^26^	Access; exchange
CMS Interoperability and Patient Access Rule (CMS)	2020 (implementation 2021+)	Required payers to offer FHIR patient access APIs; hospital event notifications; expanded patient access through portals and applications while strengthening interoperability and data exchange^27^	Access (payer API ecosystem); exchange (notifications/coordination)
Telehealth Expansion and Remote Monitoring (CMS/HHS)	2020-present (PHE flexibilities, subsequent rulemaking)	Expanded telehealth coverage and remote care; portals often used for scheduling, communication, and data sharing.^28^^,^^29^	Access; administration; exchange

This table summarizes timing and intent of policies; alignment with observed trends is descriptive, not causal. Abbreviations: CMS, Centers for Medicare & Medicaid Services; EHR, electronic health record; HHS, Department of Health and Human Services; HL7, Health Level Seven International; FHIR, Fast Healthcare Interoperability Resource; IT, information technology; MU, Meaningful Use; ONC, Office of the National Coordinator for Health Information Technology; PHE, Public Health Emergency; VDT, view/download; USCDI, United States Core Data for Interoperability.

To ensure comparability across years, we analyzed features that were consistently fielded; items introduced later (eg, external record import) or discontinued (eg, amend information after 2022) were examined only within their available windows, and trend tests were restricted accordingly. For each PE functionality within these domains, we calculated the national proportion of hospitals reporting its availability for each year from 2012 to 2024 (see Table 1 for domain definitions and policy context). Because the number of valid responses fluctuated by year and by specific survey item, the exact denominators used for these calculations are detailed in Table A1, which provides year- and measure-specific sample sizes.

### Statistical Analysis

We used the nonparametric Mann-Kendall (MK) test to evaluate the statistical significance of changes in adoption rates over time. The MK test is appropriate for time‐series data because it does not assume an underlying normal distribution.^30^^,^^31^ The direction of each trend was determined by the sign of Kendall’s τ statistic, with positive values indicating upward trends and negative values indicating downward trends. Two-sided *P* values less than .05 were considered statistically significant.

## Results

### Trends in Patient Access to Health Information

**Figure 1. Trends in Hospital Adoption of Patient Information Access Functionalities, 2012–2024:**
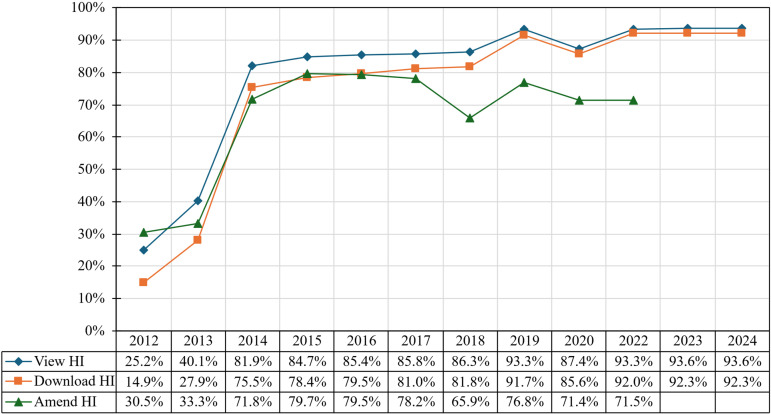
Data come from American Hospital Association Annual Survey Information Technology Supplement, 2012–2024. Adoption rates represent the national proportion of US hospitals reporting the availability of each patient engagement functionality. Comparable item-level data were not available for 2021; therefore, 2021 is excluded. HI, health information.

Figure 1 shows national adoption trends for 3 functionalities that enable patients to access their health information between 2012 and 2024. Overall, adoption increased substantially, with functionalities for viewing and downloading information reaching near-universal levels. The capabilities for patients to view and download their health information followed nearly identical trajectories of rapid growth and subsequent market saturation. In 2012, adoption rates were 25.2% for viewing and 14.9% for downloading. Both surged through 2014, reaching 81.9% and 75.5%, respectively, and continued to grow more modestly thereafter, with 92% of hospitals adopting both functionalities by 2024. The MK test indicated statistically significant and strong positive monotonic trends over the study period (viewing: τ = 0.94; *P* < .001; downloading: τ = 0.97, *P* < .001).

By contrast, the functionality allowing patients to amend their health information showed a different pattern. Adoption increased rapidly from 30.5% in 2012 to a peak of 79.5% in 2016 but then declined to 65.9% by 2018 and stabilized around 70% through 2022. The AHA IT Survey discontinued this measure after 2022. Given this volatility, the MK test did not identify a statistically significant monotonic trend over the 10-year period (amend information: τ = 0.11; *P* = .72).

### Trends in Online Administrative Services

**Figure 2. Trends in Hospital Adoption of Online Administrative Services, 2012–2020:**
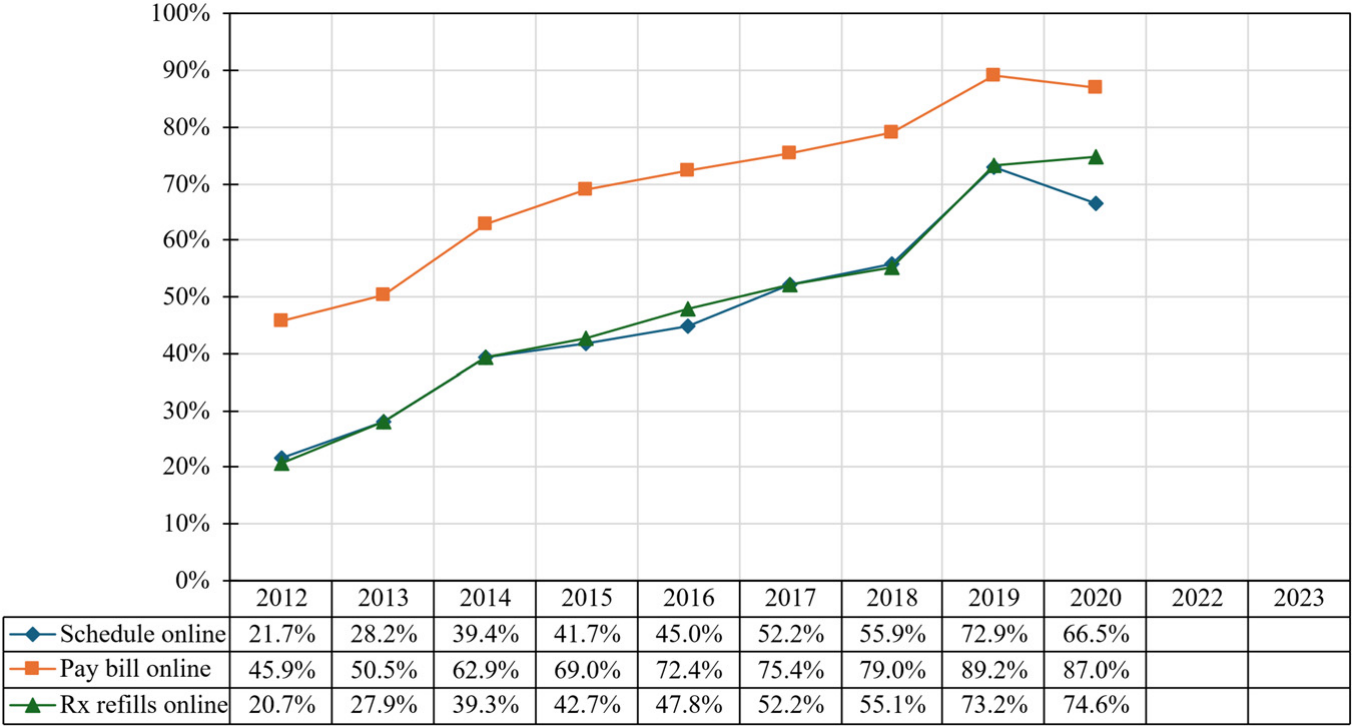
Data come from American Hospital Association Annual Survey Information Technology Supplement, 2012–2020. Adoption rates represent the national proportion of US hospitals reporting the availability of each patient engagement functionality. Items were not fielded after 2020.

Figure 2 presents national adoption trends for online administrative services, which demonstrated consistent growth across all 3 measured functionalities from 2012 to 2020. The most widely adopted tool was the ability for patients to pay bills online, which increased from 45.9% in 2012 to 87.0% in 2020, surpassing 75% by 2017. The MK test indicated a highly significant and strong positive monotonic trend (online bill payment: τ = 0.94; *P* < .001).

The functionalities for scheduling appointments online and requesting prescription refills online began at much lower adoption levels—21.7% and 20.7%, respectively—but exhibited rapid and steady growth. Both exceeded 65% by 2020, with prescription refills outpacing scheduling in the final year (74.6% vs 66.5%). Statistical analysis confirmed significant monotonic increases across the study period (scheduling: τ = 0.94, *P* < .001; prescription refills: τ = 1.00, *P* < .001).

### Trends in Patient-Provider Information Exchange

**Figure 3. Trends in Hospital Adoption of Patient-Provider Information Exchange Functionalities, 2012–2024:**
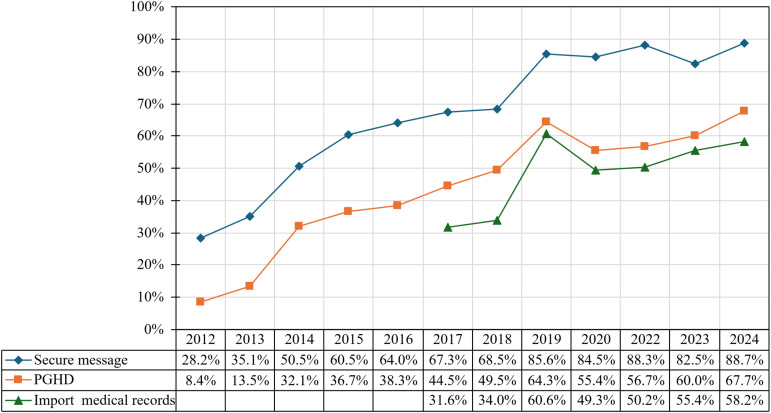
Data come from American Hospital Association Annual Survey Information Technology Supplement, 2012–2024. Adoption rates represent the national proportion of US hospitals reporting the availability of each patient engagement functionality. Comparable item-level data were not available for 2021; therefore, 2021 is excluded. PGHD, patient-generated health data.

Figure 3 illustrates adoption trends for functionalities supporting information exchange between patients and providers. Secure messaging experienced the largest increase, rising from 28.2% in 2012 to 88.7% in 2024, making it the most widely adopted tool in this domain. Similarly, the ability for patients to submit PGHD grew from 8.4% in 2012 to 67.7% in 2024. Both functionalities showed a temporary decline in 2020 but recovered in subsequent years. The MK tests confirmed statistically significant and strong positive monotonic trends (secure messaging: τ = 0.88; *P* < .001; PGHD submission: τ = 0.91; *P* < .001).

The most recent functionality—allowing patients to import external medical records—was first tracked in 2018. Adoption increased from 34.0% in 2018 to 60.6% in 2019, declined to 49.3% in 2020, and then recovered gradually to 58.2% by 2024. Because of these fluctuations over a relatively short time frame, the MK test did not detect a statistically significant monotonic trend (import medical records: τ = 0.62; *P* = .072).

## Discussion

This study examined national trends in hospital adoption of PE functionalities between 2012 and 2024 using AHA IT Supplement data. Overall, adoption increased substantially across multiple portal-related capabilities, with viewing and downloading health information reaching near-universal levels by the end of the study period. Online administrative services, including bill payment, appointment scheduling, and prescription refills, also demonstrated strong growth over their measurement window. Similarly, patient-provider information exchange functionalities expanded markedly, particularly secure messaging and the submission of PGHD. In contrast, selected advanced capabilities showed less consistent trajectories: the ability for patients to amend their health information increased early but later declined and stabilized before being discontinued in the survey after 2022, and the ability to import external medical records exhibited fluctuation after it was first fielded in 2018.

This temporary decline and fluctuation observed around 2020 may reflect the impact of the COVID-19 pandemic, during which hospitals rapidly shifted priorities toward acute care delivery and emergency response, potentially delaying the implementation or expansion of certain PE functionalities.

Collectively, these patterns indicate broad national progress in the availability of PE functionalities, while also highlighting variability across specific features and measurement periods. Taken together, these trends suggest that patient portals have transitioned from optional engagement tools to core infrastructure supporting access, communication, and administrative interaction in digitally enabled health systems.

These adoption trends in PE functionalities closely align with the timeline of major federal health IT policies implemented over the past decade. The rapid rise and near-universal uptake of functionalities for viewing and downloading health information coincide with the early stages of the MU program, which required hospitals to provide patients with timely electronic access to their records. Continued growth in online administrative services and patient-provider communication tools parallels the transition to PI, which shifted policy emphasis toward interoperability and patient-centered engagement. More recently, expansion of information exchange functionalities, particularly secure messaging and PGHD submission, is consistent with the policy environment shaped by the 21st Century Cures Act and its information-blocking provisions, which strengthened expectations for electronic access and data sharing. The inconsistent adoption of more advanced functionalities (eg, allowing patients to amend the records) may reflect ongoing challenges related to interoperability, workflow integration, governance, and perceived clinical value rather than lack of policy attention. Importantly, these findings describe national adoption patterns and their temporal alignment with federal policy milestones; they do not establish causal effects of specific policies on adoption.

Our findings are consistent with prior research documenting substantial growth in portal availability over the past decade, alongside evidence that advanced features can be more difficult to implement and sustain.^1^^,^^2^^,^^8^^,^^10–12^ Earlier studies reported widespread portal availability by the mid-2010s but with variation in the breadth of functionalities supported and operational challenges associated with more complex capabilities.^1^^,^^2^^,^^8^^,^^9^ For example, national descriptive analyses across multiple data sources have similarly shown dramatic increases in electronic health record adoption and patient access to online health information, including through patient portals, as part of broader health IT progress in the United States.^12^

By demonstrating longitudinal, hospital-level adoption patterns across multiple engagement domains, this study adds national context regarding which functionalities have reached saturation and which continue to show variability during the study period. By examining more than a decade of national hospital-level data across multiple engagement domains, this study provides one of the most comprehensive longitudinal assessments of patient portal functionality adoption within the US health IT policy context.

### Implications for Policy and Practice

The findings from this study underscore the critical role of federal health IT policy in shaping the adoption of PE functionalities in US hospitals. MU, PI, and the 21st Century Cures Act collectively created strong incentives for hospitals to adopt and expand patient portal capabilities. The near-universal availability of core tools, such as viewing and downloading health information, demonstrates the effectiveness of these policies in establishing a national baseline standard for patient access. As a result, patient portals have transitioned from optional engagement tools to core components of hospital digital infrastructure.

At the same time, variability in the adoption of more advanced functionalities, such as the ability to amend records or import external medical information, suggests that the US health system has entered a new phase of patient portal maturity. In this phase, the primary challenge is no longer initial adoption but optimization, integration, and sustained engagement. Future policy initiatives should move beyond ensuring portal availability to promoting meaningful use, equitable access, and integration of portals into broader strategies for patient-centered care and health equity. Emerging evidence indicates that even when access requirements are met, patients may continue to face usability-, literacy-, and workflow-related barriers, underscoring the importance of focusing on implementation quality rather than availability alone.^17^

Availability does not guarantee effective use or equitable benefit. Hospitals serving rural and underresourced communities often face technical, staffing, and infrastructure constraints, whereas patient-level disparities related to race, income, language, and digital literacy persist. Although this study does not examine adoption by hospital or patient characteristics, prior research suggests that inequities in portal use frequently emerge during implementation and sustained use rather than at the point of initial availability.

From a practice perspective, the near-universal adoption of core access functionalities indicates that hospitals have largely met baseline policy requirements. The next phase of improvement should emphasize usability, workflow integration, governance, and ongoing performance monitoring to ensure that patient portals function as active tools for engagement rather than as passive repositories of information.

Hospitals may benefit from dedicated governance structures for PE technologies, interdisciplinary collaboration across clinical, IT, and health information management teams, and routine evaluation of portal performance beyond simple availability. In addition, aligning portal tools with routine care delivery—such as establishing response-time standards for secure messaging and integrating scheduling and prescription refill workflows—may help reduce administrative burden for both patients and staff and strengthen meaningful engagement. From a systems perspective, effective portal implementation requires alignment across technology, workflow, governance, and workforce capacity; without coordinated strategies, hospitals risk maintaining portals that meet regulatory requirements but fall short of supporting meaningful PE.

### Limitations

Several limitations should be considered when interpreting these findings. First, the study relies on self-reported data from the AHA IT Supplement, which may be subject to reporting bias; because participation varied by year, unobserved differences between respondents and nonrespondents may also influence estimated adoption rates. Second, the survey instrument evolved over time, so not all functionalities were tracked consistently across the study period (eg, amending information was discontinued after 2022, and external record import was introduced in 2018). Third, our analysis captures hospital-reported availability of functionalities rather than patient-level use or quality of engagement. Fourth, although the MK test is appropriate for identifying monotonic trends, it does not account for short-term fluctuations or more complex dynamics. Finally, this study is descriptive and does not establish causal relationships between federal policy initiatives and adoption. Although observed trends aligned with the timing of MU, PI, and the 21st Century Cures Act, other factors (eg, consumer demand, market competition, and technological advances) likely contributed.

### Future Research

Future research should build on these findings by linking hospital-level adoption with patient-level engagement and outcomes. Integrating AHA IT Supplement data with other sources, such as the Health Information National Trends Survey or clinical outcomes data sets, could provide richer insights into how portal functionalities translate into actual patient use and improvements in care. Additional work is needed to examine disparities in portal adoption and engagement, with particular attention to rural hospitals, safety-net institutions, and populations historically underrepresented in digital health use. Importantly, future studies should move beyond descriptive analyses to explore causal relationships between federal policies and hospital adoption of PE tools. Quasi-experimental designs, such as interrupted time-series analyses or difference-in-differences approaches, may be particularly useful for assessing the direct impact of MU, PI, and the 21st Century Cures Act on hospital practices. Finally, as new technologies such as mobile applications, APIs, and artificial intelligence-enabled tools continue to evolve, future studies should assess how these innovations interact with patient portals to shape the next phase of PE in US hospitals.

## Conclusion

In summary, this study demonstrates that US hospitals made substantial progress in adopting PE functionalities between 2012 and 2024, with near-universal availability of core tools such as viewing and downloading health information and notable growth in online administrative and communication services. Although the trajectory of adoption reflects the influence of federal health IT policies, variability across functionalities and persistent disparities underscore the need for continued efforts to promote equitable and meaningful use of patient portals. Sustained policy support, coupled with innovations in technology and strategies to reduce barriers, will be essential to ensure that PE tools fulfill their potential to enhance access, communication, and patient-centered care.

## Disclosure

The authors have nothing to disclose.

## Funding

The authors received no funding for this research.

CE Quiz

## Appendix

**Table A1.** Annual Hospital Sample Sizes by Patient Engagement Functionality, 2012–2024 This table provides the year-specific valid response counts (denominators) used to calculate the national adoption rates for each patient engagement functionality. The overall sample size varies by measure and year due to item nonresponse and changes to the survey instrument over time. Data for 2021 are excluded because the American Hospital Association Information Technology Supplement was not conducted that year. Abbreviations: HI, health information; NA, not applicable; PGHD, patient-generated health data.

**Year**	**View HI**	**Download HI**	**Amend HI**	**Schedule online**	**Pay bill online**	**Rx Refills online**	**Secure message**	**PGHD**	**Import medical records**
2012	3396	3396	3396	3396	3396	3396	2153	3396	NA
2013	3223	3227	3228	3229	3228	3229	2174	3229	NA
2014	3262	3120	3202	3202	3195	3179	3170	3104	NA
2015	3456	3249	3377	3311	3354	3272	3312	3231	NA
2016	3561	3374	3486	3448	3474	3452	3474	3342	NA
2017	3463	3321	3408	3382	3388	3355	3382	3286	2990
2018	3495	3348	3431	3463	3443	3429	3451	3339	3324
2019	2941	2746	2889	2708	2968	2714	2738	2645	2623
2020	2831	2683	2798	2723	2761	2708	2448	2675	2661
2022	3111	2923	3076	NA	NA	NA	2612	2960	2936
2023	3108	NA	3087	NA	NA	NA	2736	2955	2944
2024	2580	NA	2532	NA	NA	NA	2337	2380	2413
